# Photobiomodulation use in ophthalmology – an overview of translational research from bench to bedside

**DOI:** 10.3389/fopht.2024.1388602

**Published:** 2024-08-15

**Authors:** Krisztina Valter, Stephanie E. Tedford, Janis T. Eells, Clark E. Tedford

**Affiliations:** ^1^ Clear Vision Laboratory, John Curtin School of Medical Research, Eccles Institute of Neuroscience, Canberra, ACT, Australia; ^2^ School of Medicine and Psychology, Australian National University, Canberra, ACT, Australia; ^3^ LumiThera, Inc., Poulsbo, WA, United States; ^4^ College of Health Professions and Sciences, University of Wisconsin-Milwaukee, Milwaukee, WI, United States

**Keywords:** photobiomodulation, ophthalmology, retina, age-related macular degeneration, diabetic retinopathy, retinopathy

## Abstract

Photobiomodulation (PBM) refers to the process in which wavelengths of light are absorbed by intracellular photoacceptors, resulting in the activation of signaling pathways that culminate in biological changes within the cell. PBM is the result of low-intensity light-induced reactions in the cell in contrast to thermal photoablation produced by high-intensity lasers. PBM has been effectively used in the clinic to enhance wound healing and mitigate pain and inflammation in musculoskeletal conditions, sports injury, and dental applications for many decades. In the past 20 years, experimental evidence has shown the benefit of PBM in increasing numbers of retinal and ophthalmic conditions. More recently, preclinical findings in ocular models have been translated to the clinic with promising results. This review discusses the preclinical and clinical evidence of the effects of PBM in ophthalmology and provides recommendations of the clinical use of PBM in the management of ocular conditions.

## 1 Introduction

Photobiomodulation (PBM) is an established, noninvasive, light energy-based biotechnology with thousands of published articles detailing cellular mechanisms and effects in a variety of medical conditions. The field is complicated by the numerous names used to previously describe PBM (e.g., low level laser therapy, cold laser, laser bio-stimulation, light therapy) prior to its established “PBM” designation which leads to an underestimation in the exact number of studies that have been conducted in this area. PBM is a nonthermal biological process activated by specific wavelengths of light via photoacceptor molecules to induce a cascade of physiological events. These light-induced biological changes activate signaling cascades that drive cellular changes culminating in improved cellular function and clinical outcomes (1). Enzyme cytochrome c oxidase (CcO), complex IV of the mitochondrial respiratory chain, is a major intracellular photoacceptor and transducer of photosignals for red (600-750 nm) to near-infrared (750-1100 nm) (FR/NIR) light wavelengths and is considered a primary mechanism underlying PBM effect (2, 3). CcO is the terminal electron acceptor in the respiratory chain and catalyzes the transfer of electrons from cytochrome c to molecular oxygen, thereby promoting cellular bioenergetic output due to increased oxidative phosphorylation for adenosine triphosphate (ATP) synthesis (4, 5). CcO contains two copper centers (Cu_A_ and Cu_B_) and two heme centers (heme-*a* and heme-*a3*) that absorb FR/NIR light. This light-photoacceptor interaction has been hypothesized to modify the redox state of CcO increasing the electrochemical proton gradient, thereby increasing mitochondrial membrane potential (MMP), ATP, and second messenger cyclic adenosine monophosphate (cAMP) generation (6–8).

There is also evidence that FR/NIR light augments nitric oxide (NO) bioavailability by multiple mechanisms (9). FR/NIR light photo-dissociates NO from its binding site on CcO resulting in an enhancement of CcO activity. FR/NIR light also increases NO synthesis and photo-dissociates NO from hemoglobin and myoglobin. Redox changes, NO, and calcium flux activate transcription factors leading to increased gene expression (10, 11). Microarray analysis of gene expression shows upregulation of a number of genes in the retinas of rodents treated with 670 nm. Those affected include regulatory genes, such as miRNAs. PBM-induced alterations in gene transcription leads to upregulation of genes in antioxidant pathways, immune modulation, increases in mitochondrial biogenesis, changes in mitochondrial fusion and fission, and improve cell survival (12, 13). PBM influence on CcO and mitochondrial output has been documented using many types of experimental and clinical models, and in many differing tissues, supporting its proposed role as the primary mechanism underlying the beneficial effects of PBM (14–19).

Specific to the eye, photoreceptors consume more oxygen per gram of tissue weight than any other cell in the body making the retina one of the highest oxygen-consuming tissues in the entire human body (20). Photoreceptors require high levels of energy for normal functioning and high concentrations of mitochondria are present in these tissues to meet these requirements. Consequently, retinal mitochondrial health is important for photoreceptor cell function and visual output (21, 22). The intense oxidative phosphorylation in photoreceptor cell inner segments, coupled with high concentrations of polyunsaturated fatty acids in their outer segments, render the retina susceptible to oxidative stress and lipid peroxidation (23). The presence of oxidative damage initiates an inflammatory response in the retina, leading to chronic degenerative conditions (21, 24). Normally, oxidative damage is minimized by endogenous antioxidants and cellular repair systems. However, in conditions where these repair mechanisms are not fully functional, the retina is highly vulnerable to degenerative changes. For example, preterm babies present with an immature antioxidant system which renders the developing retina of these babies susceptible to oxidative damage (25). Oxygen toxicity on immature retinal tissue contributes to the development of retinopathy of prematurity (ROP), an ocular condition that causes vision loss and blindness (26). On the other hand, in the aging retina, mitochondrial function diminishes resulting in a reduction of antioxidant and repair system functions. Thereby, aging results in retinal dysfunction and cell loss due to overt oxidative challenges (27–29). Mitochondria are critical for cellular health regulation and disease progression. Mitochondrial dysfunction, oxidative damage, and inflammation are hallmarks of many degenerative diseases, including ocular conditions such as ROP, age-related macular degeneration (AMD), retinitis pigmentosa (RP), diabetic retinopathy (DR), and diabetic macular edema (DME) (21, 30–32). Dysfunction at the mitochondrial level limits cellular energy (ATP) production, increases the generation of reactive oxygen species (ROS), and propagates apoptotic pathways. Mitochondrial repair and attenuation of oxidative stress are key to the survival of the retina. The antioxidant and anti-inflammatory effect of PBM therapy is well-established (1, 7, 8). As mitochondrial dysfunction is a key contributor to the pathological underpinnings of many ocular conditions, it is logical that PBM has been proposed as a targeted treatment option for exploration in ophthalmology.

## 2 Key parameters to establish treatment protocols

**Figure 1:**
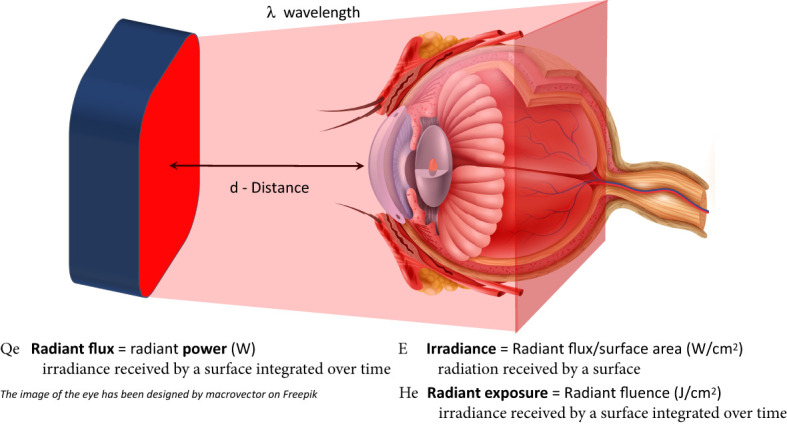
Key parameters in photobiomodulation therapy.

PBM is a complex biotechnology with a multitude of modifiable factors that can influence treatment response. Key parameters include wavelength (nm), power and energy (W/J), pulse structure (frequency (Hz), duration (s), duty cycle), irradiation time, and treatment interval. The vast number of modifiable factors introduces challenges to standardization of treatment protocols and interpretation of inconsistencies in the literature. If incorrect parameters are applied, treatment results are likely to be ineffective. Not all investigators understand the impact each parameter may have on effect. Furthermore, not all research sites have the instrumentation or trained staff to measure device output accurately, resulting in study failures. Another cause of failure occurs whenever the terms are misused or wrongly reported. Additionally, lack of precise methodologies reported in the published literature adds to the challenge. There are three major concerns regarding much of the PBM literature: 1) incomplete, inaccurate and unverified irradiation parameters; 2) miscalculation of ‘dose’; and 3) the misuse of appropriate light property terminology. **Figure 1** provides key parameters in determining PBM treatment dosage and the appropriate terminology and units that should be used in reports.

### 2.1 Wavelength

Hamblin and Demidova have previously described a wavelength range of 600-1100 nm, which falls within the ‘optical window’ (33). Chromophores, ubiquitous in tissue, including hemoglobin, oxy-hemoglobin, and melanin, absorb light in the range below 600 nm, whereas the absorption of water increases around 1200 nm. Therefore, there is an optical window in the spectral range of 600 – 1100 nm (red to infrared) when light can penetrate deep into the tissue and reach alternative photoacceptors. One such photoacceptor is CcO. The absorption spectrum for CcO closely aligns with its enzymatic activity and ATP production (34), and therefore it has been postulated to be the primary photoacceptor in PBM. Karu et al. (3) identified four absorption peaks (620, 680, 760, and 820 nm) relating to the redox-active copper centers of the enzyme. Subsequently, Wong-Riley et al. (34) found two peaks of absorption in the oxidized form of the enzyme, one at 670 nm and the second at 830 nm, with the least effective wavelength noted at 728 nm. This curve aligned with the activity of CcO. In healthy, normoxic cells, the primary activity of CcO results in increased electron transport, and consequently increased ATP production and a decrease in ROS. Following absorption of 670 nm light in primary visual cortex neurons, an increase in metabolic activity and ATP production is observed, which further supports the hypothesis of CcO being the primary target of PBM (34). Recently, Eells et al. evaluated the positive effects of 670 nm on wound healing in a diabetic mouse model using *in vivo* fluorescence imaging and showed an increased mitochondrial redox ratio and a related decrease in oxidative stress following PBM (35).

Another hypothesis considers the role of NO in controlling mitochondrial metabolism. NO inhibits mitochondrial respiration by binding to a copper center of CcO. PBM at 670 and 830 nm has shown to dissociate NO from its target, and thereby disinhibit CcO activity and restore mitochondrial metabolism. More recently, an alternative enzymatic source of cellular NO has been described, the CcO/NO activity of CcO (11, 36). This activity is reduced in normoxia but increases in hypoxia and anoxia. Following tissue injury, inflammation, hypoxia or anoxia, the redox status of the tissue changes, lowering tissue pH, which causes a switch in the function of CcO over to the CcO/NO pathway leading to an increased NO synthesis and photodissociation of NO. The presence of this switch can explain the increased NO presence after irradiation with 590+/- 14 nm of hypoxic tissue (37). The NO is released from the mitochondria, and acts as intercellular hypoxia signal, while extracellularly as a vasodilator.

The majority of research has focused on 630-670 nm and 780-850 nm light, as these wavelengths are shown to be beneficial in inflammatory conditions, wound healing, cell proliferation, and differentiation. There is evidence that a wider range of wavelengths can induce cellular changes by acting through different mechanisms. It has been postulated that wavelengths between 600-810 nm act through CcO, improving the proton gradient in terminal phosphorylation path, to produce ATP, reduce ROS, dissociate NO, and modulate Ca^2+^ signaling. Wavelengths between 800 and 1064 nm act through light- and heat-gated channels, such as the transient receptor potential (TRP) family of receptors, leading to Ca^2+^ influx into the cell and reacting with cAMP, ROS, and NO leading to upregulation of transcription factors, such as NFkB (38, 39). Further, *in vitro* and *in vivo* research has demonstrated that PBM also induces changes in gene regulation, leading to epigenetic changes and modulation of non-coding RNAs (40–44). Overall, the evidence for PBM effect across wavelengths in the 600-1100 nm range is substantial, with positive benefit attributed to multiple actions at the cellular level.

Multiwavelength versus single wavelength treatment is also a topic of interest for determination of optimized treatment protocols. Historically, single wavelength applications have been utilized in research purposes and limited to visible wavelengths. Several recent studies have investigated multiwavelength PBM in animal models of ocular conditions. Goo et al. (45) studied the *in vivo* effects of multiwavelength PBM (680, 780, and 830 nm) delivered by a light emitting diode (LED) device in a mouse model of dry eye disease (DED). Multiwavelength PBM increased the tear volume and reduced corneal irregularity. PBM inhibited corneal epithelial damage and the associated reduction in epithelial thickness, and reduced circulation of pro-inflammatory cytokines. A second study by Goo et al. (46) investigated multiwavelength PBM (680, 780, and 830 nm) in a rat model of AMD in which rats were treated with sodium iodate to induce oxidative damage to retinal tissue. PBM treatment protected the retinal tissue from oxidative damage, and reduced apoptosis of retinal and rod bipolar cells. PBM inhibited photoreceptor degeneration and reduced retinal pigment epithelium (RPE) toxicity. These studies confirm previous reports, which only use individual wavelengths, showing positive effects at the cellular level on oxidative stress, reductions in apoptotic pathways, and preservation of ocular tissue integrity (45, 46).

Recent clinical work in ophthalmology has utilized multiwavelength approaches as well (47–53). A multiwavelength approach allows for activation of differing cellular targets and likely provides synergistic functions to improve and bolster mitochondrial output. Merry et al. were the first to utilize a multiwavelength approach with two devices delivering 590, 670 and 790 nm wavelengths in subjects with dry AMD. The 670 ± 15 nm wavelength delivery specifics were 50–80 mW/cm^2^ (4–7.68 J/cm^2^) for 88 seconds (WARP10, Quantum Devices, Newark, OH, USA) and the wavelengths of 590 ± 8 nm (4 mW) and 790 ± 60 nm (0.6 mW) were delivered for 35 seconds, and pulsed at 2.5 Hz (250 milliseconds on, 150 milliseconds off) delivering 0.1 J/cm^2^/treatment (Gentlewaves, Light Bioscience, Virginia Beach, VA, USA) (48). Most recently, studies using the Valeda^®^ Light Delivery System (LumiThera, Inc., Poulsbo, WA, USA) employ a similar multiwavelength approach in one device with positive effects seen across several ocular indications (47–53). Valeda delivers three wavelengths in the yellow (590 nm; 4 mW/cm^2^; 2x 35s), red (660 nm; 65 mW/cm^2^; 2x 90s), and NIR (850 nm; 0.6 mW/cm^2^; 2x 35s) range. The 590 wavelength stimulates CcO activity and increases NO synthesis, resulting in vasodilation, and improved local oxygenation to target tissues. The 660 nm wavelength promotes O_2_ binding to the CcO active Cu_B_/Fe_a3_ site, and 850 nm drives electron transfer at the Cu_A_ site of CcO. Resulting effects include an upregulated electron transport pathway, increases in energy (ATP) production, reduced inflammation, increased cell viability, and reduced cell death by apoptosis (34, 37, 54). From a mechanistic standpoint, multiwavelength approaches may aid in optimization of treatment parameters to induce positive effects at the cellular level, ultimately driving improvements in overall cellular status. Further studies are necessary to determine the benefit of single vs. multiwavelength approaches and their impact across differing disease states and severity.

Pulsed vs. continuous wavelength waveform selection can also influence tissue penetration. Pulsed vs. continuous waveforms are considered a modifiable factor for increasing tissue penetration. A pulsed profile can increase tissue penetration compared to continuous modes with the same average power. This is attributed to increased penetration of photons at the pulsed peak. The peak power is expressed as average power divided by duty cycle. Duty cycle refers to the fraction of time the LED/Laser is on and active in its application (e.g., a continuously delivered treatment has a 100% duty cycle). The peak power, with a pulsed application and 50% duty cycle, is twice as high as a continuous application (55). The benefits of a pulsed waveform may be due to resonance frequency effects, mimicking of innate cellular process, or the direct effects on NO dissociation and binding patterns. This parameter may not be as relevant for the eye as this target tissue is easily accessible however this is a consideration for deeper tissues.

### 2.2 Accurate radiometry and dosimetry

The dose-response relationship for PBM is dependent on two parameters that determine the dose and the biological response: 1) irradiance (W/cm^2^) and 2) exposure duration (seconds). The product of these two components is the dose of light [radiant exposure] expressed as J/cm^2^. Many studies have shown that the application of lower irradiance for a longer duration is more therapeutically effective than high irradiance for a short duration despite the fact that the calculated dose (J/cm^2^) is equivalent (56, 57).

### 2.3 Biphasic dose-response curve

Numerous investigations have documented a biphasic dose response for PBM (56). At low irradiance and/or a short time of irradiation there is no response. If the irradiance is too high or irradiation time is too long, the response may be inhibited. Somewhere in the middle of this dose-response relationship is the optimal combination of irradiance and stimulation time. The PBM dose-response curve corresponds to the Arndt–Schulz curve where increasing dose corresponds to an increasing effect up to a maximum (a dose window), after which further increasing dose evokes a negative response (56–58). This biphasic dose-response curve should also be considered when interpreting negative (no effect) findings from studies with designs that utilize repeated treatment schedules within short durations of time. Further research is necessary to expand upon the optimal schedule for treatment protocols.

### 2.4 Impact of disease severity

Exposure to excessive light leads to photoreceptor damage and death. Bright white light exposure has been shown to induce a synchronized burst of apoptosis in photoreceptors in a large retinal area enabling the investigation of cellular and molecular events in a controlled fashion. For this reason, light-damage or photo-oxidative damage has been widely used to model retinal degenerative diseases including AMD (59). Light intensities in these studies are expressed as lux since the ambient light in which the rodents are housed is expressed in lux.

Two experimental studies have investigated the impact of disease/injury severity on the efficacy of PBM using this established photo-oxidative rodent model of retinal degeneration (60, 61). Qu et al. (61) examined the effects of 670 nm light irradiation in rats exposed to bright white light for 3 hours at 3 light intensities (900, 1,800, or 2,700 lux). PBM of 90 J/cm^2^ (50 mW delivered for 30 min) was delivered 3 hours prior, and then at subsequent timepoints after bright light exposure (0, 24 and 48 hours). The low intensity light (900 lux) did not cause any damage, and PBM-treated retinas did not differ histologically from control. High intensity light (1,800 lux) caused damage to the outer retina, which was significantly attenuated by PBM. In the extreme intensity of 2,700 lux caused irreversible damage to the retina, which was not attenuated by PBM. Retinal function, assessed by full field scotopic electroretinography (ERG) correlated well with the structural results.

Chu-Tan et al. (60) investigated the impact of disease/injury severity on the efficacy of PBM. They similarly conducted a progressive dosing design of light-induced retinal damage with exposure to low (750 lux), moderate (1,000 lux) and high (1,500 lux) intensity white light for 24 hours. Retinas were pre-treated with PBM irradiation, once a day for 5 consecutive days prior to light damage. They examined the effects of 4 fluences of 670 nm PBM irradiation: 9, 18, 36 and 90 J/cm^2^. In retinas exposed to the low or moderate intensity of white light, the 9 and 18 J/cm^2^ PBM fluence significantly reduced photoreceptor cell death. Treatment with 36 J/cm^2^ had no effect on retinal cell death compared to non-treated controls, whereas 90 J/cm^2^ significantly increased cell death, indicating that it was damaging to the retina. Interestingly, in retinas exposed to high intensity (1,500 lux) white light, only 90 J/cm^2^ PBM treatment showed a significant mitigation of photo-oxidative damage in the outer retina. The results of these pre-clinical studies suggest that disease severity should also be considered when establishing effective treatment doses.

Clinically, PBM poses to differentially impact disease states as the underlying mechanism of effect requires viable tissue. Patients with significant cell loss, such as AMD patients with geographic atrophy (GA), may not respond as robustly to treatment as patients with earlier stages of disease that do not show significant cell loss. This was evidenced in the LIGHTSITE I study which evaluated multiwavelength PBM effect in subjects with dry AMD. This study showed that subjects with later stages of disease that presented with GA significantly involving the foveola did not show as robust of improvements in visual acuity (VA) clinical outcomes when compared to earlier stage dry AMD subjects (49). A recent report by Franceschelli et al. (62) also demonstrates significant improvements in VA and microperimetry following 630 nm PBM treatment in severe dry AMD with macular atrophy sparing the foveola. This was further evidenced in a recent study report by Romero et al. (63) who showed significant improvements in VA following multiwavelength PBM treatment in AMD subjects with no atrophy compared to no change in AMD subjects with foveal or extrafoveal atrophy. While PBM effect in later stage patients may not show robust improvements in clinical outcomes, the potential for preservation of the current visual status is also of importance when considering degenerative disease states in the eye.

Future studies would be of value to assess outcomes across stages of disease to evaluate effect expectations for improvements vs. preservation of current visual output. This will also aid in aligning patient expectations with realistic outcomes.

## 3 Evidence in experimental and clinical disease

Growing interest in the utility of PBM in ocular disease has led to the development of clinical devices designed specifically for the eye space that have been reliably tested and engineered for larger sponsor-led trials and commercial use as well. Clinical trials are generally conducted by pharmaceutical/biotechnology companies with the significant financial resources needed to complete these widescale evaluations. While these industry-backed trials are critical for investigative research to supplement initial pilot data, the addition of reports from independent research and academic sites are invaluable to support safety and efficacy claims as well as to provide insights into the real-world effects seen outside of structured trial designs. The number of published reports in the use of PBM in the eye continues to grow and includes a number of case reports, small study designs, and RCTs. Single, limited number, and unpublished reports (e.g., conference paper/poster presentations) have known limitations regarding credibility and impact but provide exploratory evidence for further studies to be designed to elucidate potential effects. While limited in nature, these studies provide further excitement for PBM effect in other ocular indications and are mentioned in subsections as exploratory findings for consideration. Published preclinical and clinical data are discussed by indication in the subsequent sections. An overview of published clinical reports with specific details on PBM parameters used, study design, and findings are presented in **Table 1**.

**Table 1:** Clinical studies evaluating photobiomodulation in ophthalmology.

Article (First Author, year)	Study Quality*	Indication	Sample Size^	λ (nm) and Dose (J/cm2 and/or mW/cm^2^)^	PBM Specifications and Tx Design^	Key Findings
Franceschelli S. et al., 2024 (62)	1	Severe Dry AMD	60 subjects PBM group: 33 subjects (50 eyes) Control group: 27 subjects (28 eyes)	630 nm (15 μW; 2.5 μJ/cm²) Control: No LED	Frequency: PBM Tx 10x for 10 minutes/2 weeks; Assessments conducted after 10^th^ Tx	• Improved VA • Improved microperimetry (mean sensitivity) • No structural change via OCT or safety concerns
Zhou W. et al., 2024 (64)	1	Myopia	200 subjects (50 subjects for each group)	650 nm: 3 interventional levels tested - 0.37 ± 0.02 mW; 0.60 ± 0.2 mW; 1.20 mW Control: SVS only	Frequency: PBM Tx 2x per day for 3 minutes with at least a 4-hour interval between sessions for 6 months	• No progression of myopia • Reduction in SER progression • Reduced AL • Increased SFCT • No AEs
Zhou L. et al., 2023 (65)	1	Myopia	Two consecutive cohorts of 50 subjects PBM group: 25 subjects Control group: 25 subjects	650 nm ± 10 nm (0.35 ± 0.02 mW/cm2); illumination of approximately 400 lux (LD-A, Jilin Londa Optoelectronics Technology) Control: SVS only	Frequency: PBM Tx 2x per day for 3 minutes with at least a 4-hour interval between sessions for 12 months	• Stabilization of myopia progression • Reduced AL • Reduction in SER progression • No significant change in CCP, ACD, and SFCT • Most common AE included immediate, and reversible, vision loss due to flash blindness or afterimage • No severe, systemic, or other AE related to grades of ocular diseases
Boyer D. et al., 2023 (50)	1	Dry AMD	100 subjects (144 eyes) PBM group: 93 eyes Control group: 54 eyes	590 nm (5 mW/cm^2^); 660 nm output (65 mW/cm^2^); 850 nm (8 mW/cm^2^) Control: 10-100x reduction in 590 nm and 660 nm, removal of 850 nm [Valeda Light Delivery System, LumiThera, Inc]	Frequency: PBM Tx series 3x per week/3-4 weeks at BL; repeated Tx series at 4, 8, 12, 16, and 20 months	• Improved VA • No increase in drusen volume • Reduced new onset of GA • 4 ocular-specific AEs considered related to the Tx (none led to study discontinuation and were mild or moderate in intensity); No SAE considered related to Tx; no signs of phototoxicity or structural damage via OCT
Burton B. et al., 2023 (51)	1	Dry AMD	44 subjects (53 eyes) PBM group: 29 eyes Control group: 15 eyes	590 nm (5 mW/cm^2^); 660 nm output (65 mW/cm^2^); 850 nm (8 mW/cm^2^) Control: 10-100x reduction in 590 nm and 660 nm, removal of 850 nm [Valeda Light Delivery System, LumiThera, Inc]	Frequency: PBM Tx series 3x per week/3-4 weeks at BL; repeated Tx series at 4 and 8 months	• Improved VA • No increase in drusen volume • Reduced new onset of GA • No safety concerns or signs of phototoxicity observed
Kim JE. et al., 2022 (66)	1	DME	135 subjects PBM group: 69 subjects Control group: 66 subjects	670 nm Control: Broad spectrum white light	Frequency: PBM Tx 2x daily for 90 seconds for 4 months	• Reduced central subfield thickness and VA letter loss compared to control • 8 AEs possibly related to PBM device; 2 AEs possibly related to control device; No serious AEs
Jiang Y. et al., 2021 (67)	1	Myopia	264 subjects PBM group (plus SVS): 119 subjects Control group (SVS only): 145 subjects	650 nm (1600 lux and a power of 0.29 mW for a 4-mm pupil) [Eyerising, Suzhou Xuanjia Optoelectronics Technology] Control: SVS only	Frequency: PBM Tx 2x per day for 3 minutes with at least a 4-hour interval between sessions, 5 days per week, for 12 months	• Reduction in myopia progression • Reduced axial length • Reduction in SER progression • No severe AEs or structural damage to photosensory layer observed by OCT
Xiong F. et al., 2021 (68)	1	Myopia	229 subjects PBM group: 74 subjects (74 eyes) OK lens Group: 81 subjects (81 eyes) Vision distance spectacle Group: 74 subjects (74 eyes)	650 nm (2 ± 0:5mW) [Ya Kun Optoelectronic] Controls: OK lens; Vision distance spectacles	Frequency: PBM Tx 2x per day for 3 minutes with at least a 4-hour interval between sessions, for 6 months	• Reduction in myopia progression • Reduced AL • Reduction in SER progression • Increased SFCT
Markowitz S. et al., 2020 (49)	1	Dry AMD	30 subjects (46 eyes) PBM group: 24 eyes Control group: 22 eyes	590 nm (5 mW/cm^2^); 660 nm output (65 mW/cm^2^); 850 nm (8 mW/cm^2^) Control: 10-100x reduction in 590 nm and 660 nm, removal of 850 nm [Valeda Light Delivery System, LumiThera Inc.]	Frequency: PBM Tx series 3x per week/3 weeks at BL; PBM Tx series repeated at 6 months, 12-month study duration	• Improved VA • Improved contrast sensitivity • Improved QoL • Improved microperimetry (fixation stability) • Reduced central drusen volume • Reduced central drusen thickness • No AEs related to Tx
Kent AL. et al., 2020 (69)	1	ROP	86 subjects (neonates) PBM group: 45 subjects Control group: 41 subjects	670 nm (9 J/cm^2^) Control: No light	Frequency: Full body PBM Tx daily until 34 weeks of age or transfer	• No difference in severity of ROP or requirement for laser treatment • Improved survival rates • No AEs
Koev K. et al., 2017 (70)	2	AMD	55 subjects (109 eyes) PBM group: 66 eyes Control group: 44 eyes	633 nm (He-Ne Laser continuous emission at 0.1 mW/cm^2^) Control: Mock Tx	Frequency: PBM Tx 6x for three minutes once every other day for 5 years	• Improved VA • Improved VA optotypes • Reduced metamorphopsis and scotomas • In nAMD, reduced edema and bleeding
Ivandic BT, & Ivandic T., 2012 (71)	2	Amblyopia	178 subjects (231 eyes) PBM group: 211 eyes Control group: 20 eyes	780 nm (292 Hz, 1:1 duty cycle, AVG power 7.5mW; 3mm^2^) Control: No light; audible signal every 10s	Frequency: PBM Tx 4x/2 weeks	• Improved VA • Increased m-VEP amplitude • No local or systemic AEs
Ivandic BT, & Ivandic T., 2008 (72)	2	AMD	203 subjects (348 eyes) PBM group: 328 eyes Control group: 20 eyes	780 nm (292 Hz, 1:1 duty cycle, AVG power 7.5mW; 3mm^2^) Control: No light; audible signal every 10s	Frequency: PBM Tx 4x/2 weeks	• Improved VA (in eyes with and without cataracts) which was maintained for 3-36 months • Reduced metamorphopsia, scotoma, and dyschromatopsia • Reduced edema and bleeding in nAMD • No AEs
Tang J. et al., 2014 (73)	3	DME	4 subjects (8 eyes) PBM group: 4 eyes Control group: 4 eyes	670 nm (50-80 mW/cm^2^) [WARP 10, Quantum Devices] Control: Fellow eye	Frequency: PBM Tx 2x daily/2-9 months	• Reduced macular edema • Reduced focal retinal thickness • No AEs
Benlahbib M. et al., 2023 (53)	4	AMD	20 Eyes	590 nm (5 mW/cm^2^); 660 nm output (65 mW/cm^2^); 850 nm (8 mW/cm^2^) [Valeda Light Delivery System, LumiThera, Inc]	Frequency: PBM Tx 2x per week/5 weeks	• Improved VA • Reduced retinal sensitivity • Increased fixation stability • Reduced drusen volume • Reduced drusen thickness • Increased GA lesion area • Improved QoL • 2 AEs reported: A rupture of drusenoid pigment epithelial detachment 6 months post-PBM Tx and vitreous floaters
Kaymak H. et al., 2023 (47)	4	DME	18 subjects (28 eyes)	590 nm (5 mW/cm^2^); 660 nm output (65 mW/cm^2^); 850 nm (8 mW/cm^2^) [Valeda Light Delivery System, LumiThera, Inc]	Frequency: PBM Tx series 3x per week/3-4 weeks	• Reduction in CRT • Resolution of inner retinal fluid and hard exudates • Improved DRSS scores • Improved QoL • Maintained VA • No ocular or systemic AEs
Le HM. et al., 2022 (74)	4	Reticular Pseudodrusen (RPD)	5 subjects (5 eyes)	590 nm (5 mW/cm^2^); 660 nm output (65 mW/cm^2^); 850 nm (8 mW/cm^2^) [Valeda Light Delivery System, LumiThera, Inc]	Frequency: PBM Tx 2x/week for 6 weeks	• Changes in RPD distribution: Reduced number of stage 3 RPD with accompanying increase in number of stage 1 RPD • Maintained VA • No AEs
Casson RJ. et al., 2022 (75)	4	RP	12 Subjects (12 eyes)	670 nm (25 mW/cm^2^ or 100 mW/cm^2^)	Frequency: PBM 2x weekly for 8 weeks	• Improved VA • Cone-derived photopic flicker responses were almost completely abolished • No change in ERG amplitude • No AEs
Scalinci SZ. et al., 2022 (76)	4	Stargardt Disease	45 Subjects (90 eyes)	650 nm (10 Hz)	Frequency: PBM Tx 2x daily/5 days/week for 12 months	• Improved VA • Improved pERG • Improved microperimetry
Siqueira RC. et al., 2021 (77)	4	AMD	10 Subjects	670 nm (50-80 mW/cm^2^) [WARP 10, Quantum Devices]	Frequency: PBM Tx 9x	• Improved VA • Improved visual field function • No change or abnormalities in OCT, ERG, FR and AF • No AEs
Shen W. et al., 2020 (78)	4	Center-involving DME	21 Subjects	670 nm laser (3 interventional levels tested - 25, 100 or 200 mW/cm^2^)	Frequency: PBM Tx 12x/5 weeks	• Dose dependent reduction in central macular thickness • No AEs
Grewal MK. et al., 2020 (79)	4	AMD and normal ageing	31 Subjects with iAMD; 11 Subjects aged 55 years or above with normal retina	670 nm (40 mW/cm^2^ or 4.8J/cm^2^)	Frequency: PBM Tx for 2 minutes daily for 12 months	• In normal ageing, an improvement in scotopic thresholds in the group with no AMD • No significant improvement in any functional or structural changes • No effect on intermediate AMD • No serious AEs related to the device
Merry G. et al., 2017 (48)	4	Dry AMD	22 Subjects (42 Eyes)	670 nm (50-80 mW/cm^2^) [WARP 10, Quantum Devices] 590 nm (4 mW/cm^2^); 790 nm (0.6 mW/cm^2^) [Gentlewaves, Light Bioscience]	Frequency: PBM treatment series 9x/3 weeks	• Improved VA • Improved contrast sensitivity • Reduced central drusen volume • Reduced central drusen thickness • No AEs
Kent AL. et al., 2015 (80)	4	ROP	28 Subjects (56 eyes)	670 nm (9 J/cm^2^)	Frequency: PBM Tx for 15 minutes daily from ≤ 48 hours post-birth until 34 weeks postmenstrual age	• No skin burns or other documented AEs • No safety concerns
Sachdev A., 2024 (52)	5	CSCR	1 Subject (1 eye)	590 nm (5 mW/cm^2^); 660 nm output (65 mW/cm^2^); 850 nm (8 mW/cm^2^) [Valeda Light Delivery System, LumiThera, Inc]	Frequency: PBM Tx series 3x per week/3 weeks; PBM Tx series repeated at 6 months	• Improved VA • Resolution of edema
Ahadi M. et al., 2022 (81)	5	DME	1 Subject (2 eyes)	670 nm (50-80 mW/cm^2^) [WARP 10, Quantum Devices]	Frequency: PBM Tx 1x daily for month 1; 3x/week for month 2; 1x/week for month 3; 1x/month afterwards	• Improved VA • Resolution of macular edema • At 16 months follow-up, VA remained stable and OCT showed no evidence of recurrence of edema
Ivandic BT, and Ivandic T., 2014 (82)	5	RP	1 Subject	780 nm (292 Hz, 1:1 duty cycle, AVG power 10mW; 3mm^2^)	Frequency: PBM Tx 2x/2 weeks	• Improved VA

*Study Quality Assessment Designations:1: Prospective, randomized, controlled study; 2: Prospective, non-randomized, controlled; 3: Retrospective, controlled study; 4: Prospective or retrospective uncontrolled study (e.g., Observational study); 5: Case report(s) with small sample size. ^PBM specifications and study design taken from published reports where described. Number of subjects and number of eyes provided where available. AE, adverse event; AF, autofluorescence; AL, axial length; AMD, age-related macular degeneration; ACD, anterior chamber depth; BL, baseline; CCP, central corneal refractive power; CRT, central retinal thickness; DME, diabetic macular edema; DRSS, diabetic retinopathy severity scale; ERG, electroretinography; FR, fluorescence retinography; GA, geographic atrophy; IPL, intense pulsed laser; m-VEP, multifocal visual evoked potential; nAMD, neovascular age-related macular degeneration; nm, nanomolar; OCT, optical coherence tomography; pERG, pattern electroretinography; PBM, photobiomodulation; QoL, quality of life; ROP, Retinopathy of prematurity; RPD, reticular pseduodrusen; SER, spherical equivalent of refraction; SFCT, subfoveal choroidal thickness; Tx, treatment; VA, visual acuity.

### 3.1 Age-related macular degeneration

Age-related macular degeneration (AMD) is the leading cause of untreatable blindness among older adults worldwide. The global prevalence of AMD is expected to reach 288 million by 2040. Increasing age is a risk factor for development of AMD, presenting a continued concern with an aging population and increasing life expectancies (83, 84). AMD is categorized into two types: atrophic AMD (dry AMD) and neovascular AMD (nAMD). The ‘wet’ form (nAMD) of the disease, which involves abnormal growth of blood vessels into the outer retina, can be managed by anti-VEGF drugs (85). However, there is a paucity of effective treatment for the more common ‘dry’ form, which involves progressive vision loss in the absence of new vessels, though a number of new therapeutic targets have been identified and are being tested (86). Recently, new drug interventions that target complement pathways have been approved for GA secondary to AMD. Studies with these agents show slowing of GA lesion growth but no effect on clinical vision endpoints (87).

Visual loss in AMD is due, in large part, to age-dependent impairment of RPE function. Although, the pathogenesis of AMD has not been fully elucidated, considerable evidence supports a role for mitochondrial dysfunction, oxidative stress, and activation of the innate immune system in its onset and development (29, 88, 89). Approximately 60% of AMD cases are linked with a dysfuntional form of a regulator of the complement system, a component of innate immunity. The presence of uncontrolled immune activation may contribute to the progressive nature of the disease (90).

#### 3.1.1 Preclinical evidence

Albarracin et al. (91) examined the effects of 670 nm light on retinal function, photoreceptor damage, and inflammatory activation on the rodent photooxidative retinal injury model. They assessed 3 treatment paradigms of 5 treatments of 9 J/cm^2^ applied either before, during, or after bright light exposure of the retina. They report that the treatment mitigated the loss of retinal function and significantly reduced photoreceptor damage and microglia activation in all 3 treatment paradigms. Kokkinopoulos et al. (92) examined the effects of 670 nm light on retinal aging. They showed that light treatment, in doses ranging from 4-7 J/cm^2^, increases MMP and reduces retinal inflammation in the aged mouse retina. Other studies investigated the effects of PBM in a genetic model of AMD (93, 94). In the CFH knock-out model, retinal inflammation and amyloid beta deposition occur resulting in loss of retinal function. PBM-treated animals exhibit significant increases in CcO activity, and downregulation of complement component C3 in the outer retina, as well as key biomarkers of retinal stress, vimentin, and glial fibrillary acidic protein (GFAP). Notably, in the study by Begum et al. (93), 670 nm PBM irradiation was delivered by LED arrays mounted on the sides of the animal cages (360 seconds at a fluence of 7.2 J/cm^2^ twice per day for 14 days) rather than directly focused on the retina. These results suggest the abscopal effects of PBM. Calaza et al. (94) using 670 nm PBM delivering 3.6 J/cm^2^ at each treatment (40 mW/cm^2^ for 90 seconds daily for 5 days), found a significant increase in ATP production in the retina and brain of aged (8 months) CFH knock-out mice. The authors demonstrate a decline in retinal ATP concentrations prior to the appearance of the ocular phenotype at 12 months in this model, suggesting that the underlying pathomechanism may be linked to a reduction in mitochondria bioenergetics. These studies suggest that this bioenergetic dysfunction can be corrected by PBM. These studies demonstrate that a great range of overall doses, between 18-200 J/cm^2^, delivered over 1-2 weeks can be effective in mitigating degenerative changes, however, 670 nm light treatment did not reduce the Aβ load in the retina. De Taboada et al. (95) showed a reduction in Aβ plaque deposition in a mouse model of Alzheimer’s disease (AD) following 808 nm light delivered transcranial laser therapy, demonstrating different cellular and molecular targets to discrete wavelengths of light. Similarly, Di Paolo showed a positive PBM effect following exposure to bright light (1000 lux) on retinal neurodegenerative processes, preservation of retinal thickness, and reductions in gliosis and microglia invasion (96). Most recently, Goo et al. (46) saw benefit of multiwavelength PBM (680, 780, and 830 nm) in a rat model of AMD which included preservation of retinal tissue from oxidative damage, inhibition of photoreceptor degradation, reduced apoptosis of retinal and rod bipolar cells, and reduced RPE toxicity.

#### 3.1.2 Clinical evidence

A number of clinical studies have been conducted to evaluate the effect of PBM in AMD. The first of these was reported by Ivandic and Ivandic (72). This study examined 348 eyes of 203 patents with various stages of AMD, with and without cataracts present. For the control group, 20 eyes received a sham treatment. Transconjunctival laser radiation of 780 nm was delivered by a continuous emission, semi-conductor diode laser. Subjects were treated 4x over 2 weeks, delivering 1.2 J/cm^2^ fluence of irradiation overall. The authors report significant improvement compared to baseline in all treated eyes irrespective of the presence or absence of cataracts and significant improvement compared to the sham control group. In addition, the study found improvement in color vision (assessed by Farnsworth D-15), reduced metamorphopsia, size of relative scotomas, pigment accumulation and cystic drusen. In wet AMD subjects, there was a reduction in size of edema and bleeding. Subjects were followed for 7 years, and results show that the visual functional improvement was maintained for up to 36 months. No impact of cataracts was observed on the PBM benefit in any clinical outcome assessed.

Koev et al. (70) evaluated PBM in subjects with all stages of AMD with a total of 66 PBM-treated eyes and 44 control eyes. Progressive, exudative AMD (wet AMD) was diagnosed in 8 treatment eyes. Subjects were treated with a He-Ne Laser with continuous emission at 633 nm (0.1 mW/cm^2^). The treatment protocol was 6x for 3 minutes once every other day for 5 years. Improvements in VA was noted in 93.9% of subjects at the end of study (i.e., 5 years). In most cases, improvement in VA was accompanied by a decrease in metamorphopsis and scotomas. In the eyes with a wet AMD diagnosis, a reduction is edema and bleeding were noted. They also observed diminished pigment accumulation and cystic drusen (70).

Siqueira et al. (77) report on an open label, non-randomized prospective study including 10 subjects with advanced GA, with vision less than 20/100. Subjects were treated with 9 treatments of 670 nm irradiation delivered over 3 weeks with an overall fluence of 36 J/cm^2^. Visual function, visual field, VA, and structure were examined 1, 4, and 16 weeks after PBM. Baseline measures of the same eyes were used as control. The study found significant improvement after 1 week in VA and perimetry, and 80% of subjects reported subjective visual improvement. The beneficial effects were unchanged through the experimental period. No adverse events (AEs) were observed.

Grewal et al. (79) conducted a single center, pilot study with 23 subjects with intermediate dry AMD, and used healthy aging retinas as controls. Some retinas featured subretinal drusenoid deposits (n=8), others did not (n=15). They used a custom-built hand-held LED light source, emitting 650-700 nm light at 40 mW/cm^2^. Subjects treated themselves every morning for 2 minutes (4.8 J/cm^2^) for 12 months, delivering in total up to 1,752 J/cm^2^. Subjects were examined at 1, 3, 6, and 12 months by testing VA, ERG, perimetry, and imaging by optical coherence tomography (OCT). The study found an improvement in scotopic threshold in healthy eyes, but unlike other research studies, no improvements in any of the other measures assessed were observed. This failure to achieve any benefits may be the consequence of the overall high dose, and further supports earlier findings of a biphasic PBM response (57).

The initial investigation for multiwavelength PBM effect in the eye was conducted by Merry et al. (48) in the Toronto and Oak Ridge Photobiomodulation (TORPA) trial. The study included 42 eyes with dry AMD (AREDS 2-4) and no choroidal neovascularization, no cataract, or active wet AMD. The study utilized two commercial products for transpupillary treatment of 670 nm (WARP 10, Quantum Devices), and 590 nm and 790 nm (Gentlewaves, Light Bioscience) irradiation. Subjects were treated 3x every week for 3 weeks (9 treatments total). They received 4-7.68 J/cm^2^ 670 nm irradiation. Gentlewaves delivered irradiation in a pulse mode at 2.5 Hz frequency, for 35 second, with 4 mW radiant flux of 590 nm (0.14 J/cm^2^) and 0.6 mW of 790 nm (0.021 J/cm^2^) emission. The study demonstrated significant improvement of VA and contrast sensitivity (CS). They also found anatomical improvement with a significant reduction of drusen volume and central drusen thickness (48).

The recent LIGHTSITE series of clinical trials evaluating PBM in dry AMD are the most rigorously designed and controlled trials. The LIGHTSITE series of RCTs evaluate the effects of multiwavelength PBM (590 nm, 5 mW/cm^2^; 660 nm, 65 mW/cm^2^; 850 nm; 8 mW/cm^2^) using the LumiThera Valeda^®^ Light Delivery System [Valeda] in subjects with dry AMD. One series of treatment comprised of 9 sessions delivered over 3-5 weeks. Randomized sham treatment was provided by a reduced dose of selected wavelengths 590 nm and 660 nm at 10 and 100x attenuated fluence, respectively, and omission of the NIR wavelength. Markowitz et al. (49) report on the results of the LIGHTSITE I trial which enrolled 46 eyes with dry AMD (AREDS category 2-4), and best-corrected visual acuity (BCVA) of 20/40-20/200. Subjects received 2 series of PBM treatment over the course of 1 year delivered 6 months apart. The study found significant improvement in BCVA, CS, and microperimetry in PBM-treated eyes compared to sham-treated eyes. In addition, a significant reduction in central drusen volume and drusen thickness was evidenced.

The LIGHTSITE II study, reported by Burton et al. (51), enrolled 53 dry AMD eyes of 44 subjects. Enrolled subjects had intermediate stage dry AMD and baseline vision between 20/100 and 20/32 with no central-involving GA. The treatment paradigm followed the earlier trial, but here, each PBM series was repeated at 4-month intervals versus 6-month intervals in the LIGHTSITE I study. This change in study design followed the benefit profile of LIGHTSITE I study which showed a waning effect of PBM benefit after 6 months (i.e., re-treatment interval was moved up to 4 months to maintain beneficial effects). Due to the COVID pandemic, the study was truncated and only 29 eyes received the full protocol set of PBM treatments with significant disruption to the approved protocol. Nonetheless, PBM-treated eyes showed a significant increase in BCVA, confirming earlier findings and showed statistically significant improvement in BCVA at 9 months. Furthermore, they found that macular drusen volume and drusen thickness did not change over the period of study in PBM-treated eyes, while there was an increase observed in sham-treated eyes. A reduction in GA lesion area growth in PBM-treated eyes compared to sham was noted.

The LIGHTSITE III study used the same treatment paradigm as the LIGHTSITE II trial, delivering a PBM series of 9 treatments over 3-5 weeks, repeated every 4 months, for a total duration of 24 months. Boyer et al. (50) evaluated the effects of multiwavelength PBM in 148 eyes of 100 subjects making this the largest randomized control trial evaluating PBM in dry AMD to date. At the 13-month timepoint, PBM-treated eyes show a significant improvement in BCVA (mean 5.4 ETDRS letters) compared to the sham group and a reduction in disease progression to new GA. The LIGHTSITE III study is complete (97), and results further report a sustained benefit in BCVA out to 24 months (mean 5.9 ETDRS letters) and slowing of disease progression with only ~7% of the PBM group progressing to new GA versus 24% of the sham group. Loss of vision was higher in the sham group versus the PBM group suggesting early to intermediate treatment of dry AMD patients with PBM is valuable in vision preservation and disease management.

The benefit of multiwavelength PBM using Valeda is independently noted in the findings from Benlahbib et al. (53) who investigated the effects of multiwavelength PBM (590 nm, 5 mW/cm^2^; 660 nm, 65 mW/cm^2^; 850 nm; 8 mW/cm^2^) delivered 2x per week/5 weeks in 20 eyes from subjects with AMD. At 6-months post-treatment follow up, improvements in BCVA (mean 5.5 ETDRS letters), QoL, reductions in retinal sensitivity, drusen volume and thickness, and increased fixation stability were observed.

Most recently, Franceschelli et al. (62) evaluated the effect of 630 nm PBM (15 μW; 2.5 μJ/cm²) treatment in 60 severe dry AMD subjects with a macular atrophic area and relative sparing of the foveal region. Subjects received 10 PBM or control treatments delivered over 2 weeks totaling 10 minutes each. Assessments were conducted after the 10^th^ treatment and included OCT, VA, and microperimetry. Following PBM treatment, significant improvements in VA and microperimetry (mean sensitivity), as well as subjective improvements in the perceptions of colors (more sutured) and shapes (clearer) were observed. No structural change measured via OCT or safety concerns were reported.

#### 3.1.3 Additional evidence (exploratory)

A recent *post-hoc* evaluation of the LIGHTSITE III trial data comparing the risk of losing vision (>5 letter) and disease progression with evidence of new formation of GA demonstrates a positive benefit overtime for PBM effect. Schneiderman et al. (98) report a hazard ratio for BCVA with a >5 letter loss of 0.47, which indicates a statistically significant 53% reduction in onset of vision loss of >5 letters for PBM-treated eyes versus the sham treatment. In addition, the hazard ratio for onset of new GA was 0.27, indicating a statistically significant risk reduction of 73% to progress to new GA over the two-year study duration for those treated with PBM.

The ELECTROLIGHT study further extended the LIGHTSITE clinical trials of multiwavelength PBM in dry AMD, by retinal function analysis, using multi-luminance, fixed and chromatic ERG analysis. This study evaluated 15 subjects (23 eyes) with dry AMD in an open label, prospective, pilot clinical study. After one series of treatment with multiwavelength PBM (Valeda: 590 nm, 5 mW/cm^2^; 660 nm, 65 mW/cm^2^; 850 nm; 8 mW/cm^2^) delivered 3x/week over 3-4 weeks (total of 9 treatments), a significant increase in BCVA, CS, and multi-luminance ERG amplitudes was observed which was maintained six months following PBM (99).

Several additional studies have presented exciting data on the utility of PBM in dry AMD. Eisenbarth et al. (100) evaluated clinical outcomes following multiwavelength PBM (Valeda: 590 nm, 5 mW/cm^2^; 660 nm, 65 mW/cm^2^; 850 nm; 8 mW/cm^2^) in 41 subjects (72 eyes) with AREDS category 1-4. Assessments taken one month after PBM treatment showed significant improvements in BCVA, CS, Radner reading speed and critical print size, and macular mapping letter recognition. Romero et al. (63) studied the effects of multiwavelength PBM (Valeda: 590 nm, 5 mW/cm^2^; 660 nm, 65 mW/cm^2^; 850 nm; 8 mW/cm^2^) delivered 3x/week over 3 weeks in 43 subjects (67 eyes) diagnosed with stage 2-4 dry AMD. Baseline BCVA ranged from 15 to 85 letters. A significant improvement in BCVA of 12 ETDRS letters was noted in subjects that did not have atrophy. No effect on BCVA was observed in subjects with extrafoveal or foveal atrophy. No effect was observed in QoL as measured with the visual function questionnaire (VFQ).

### 3.2 Diabetic retinopathy/diabetic macular edema

DR and subsequent DME are common long-term complications of diabetes. In the first 10 years following a diagnosis of diabetes, DR is observed in almost all type 1 diabetic patients and > 60% of type 2 diabetic patients (101). DR occurs following chronic metabolic and inflammatory insult to neurovascular structures of the retina. Resulting damage leads to loss of function in retinal structures and visual impairment (102, 103). Although, the pathogenesis of DR is incompletely understood, a reduction in hyperglycemia has been shown to exert positive effects on the development and progression of DR. However, in many patients, maintenance of glycemic control is difficult. Key contributors to the pathological underpinnings include microvascular changes, inflammatory response, and retinal neurodegeneration. These are particularly prevalent in early stages of DR (104). Current therapies are invasive and include anti-VEGF intravitreal injections and laser therapy. Anti-VEGF therapy has shown benefit in DR patients although most patients do not demonstrate significant improvements in visual outcomes (104).

#### 3.2.1 Preclinical evidence

In cultured retinal cells, incubated in diabetes-like concentrations of glucose (30 mM), 670 nm PBM treatment (240 sec, 25 mW/cm^2^) delivering 6 J/cm^2^ attenuated oxidative stress, expression of inflammatory biomarkers, and improved survival in RGC5 (immortalized retinal ganglion) and 661W (immortalized photoreceptor-like) cells (105). In streptozotocin (STZ)-diabetic rats, 670 nm PBM attenuated diabetes-induced abnormalities of retinal function and reduced retinal ganglion cell (RGC) death. PBM also decreased retinal superoxide generation and inhibited diabetes-induced abnormalities in the ERG, RGC viability, superoxide generation, leukostasis, and expression of MnSOD and ICAM-1 *in vivo* (105).

A subsequent study conducted by Cheng et al. (106). investigated the impact of 670 nm (25 mW/cm^2^) on neural and vascular lesions characteristic of early-stage DR. PBM was delivered daily for 8 months to STZ-diabetic mice and evaluations on visual function, retinal capillary permeability, and capillary degeneration were conducted. PBM significantly inhibited the leakage, degeneration of retinal capillaries, and accompanying deficit in visual function induced by the animal model. PBM also showed an inhibitory effect on retinal mRNA levels and numbers of circulating stem cells. These preclinical findings support benefit of PBM to inhibit functional and histopathologic features seen in early-stage DR.

Saliba et al. (107) studied the effect of 670 nm PBM in pigmented diabetic mice. Their model included diabetic mice treated with an inhibitor of the antioxidant enzyme, heme oxygenase 1 (HO-1) and exposed to 670 nm light while their eyes were blocked from direct exposure of the light. Mice treated with 670 nm light showed both neuronal and vascular beneficial effects, further supporting the abscopal effects of PBM.

Nonarath et al. (108) investigated the modulation of signaling pathways by 670 nm light in Müller glial cells. Rat Müller glial cells (rMC-d1) were grown under normal (5 mM) or high (25 mM) glucose conditions and treated with a 670 nm LED array (4.5 J/cm^2^) or no light (sham) daily. A single 670 nm light treatment diminished ROS production and preserved mitochondrial integrity. A 3-day treatment with PBM educed NFκB activity and prevented the subsequent increase in ICAM-1. The ability of 670 nm treatment to prevent early molecular changes in this model system suggests that PBM treatment could mitigate early deleterious effects modulating inflammatory signaling and diminishing oxidative stress.

#### 3.2.2 Clinical evidence

The therapeutic efficacy of 670 nm PBM was initially investigated in a small study involving 4 subjects with non-center diabetic retinal edema (NCIDME) (73). This type of retinal edema does not impact vision as the center of the macula is spared, however; 25% of NCIDME patients progress to vision threatening CIDME within 2 years (109). Subjects were treated 2x daily at a fluence of 4.5 J/cm^2^ (90 sec per treatment at 50 mW/cm^2^) using a small handheld 670 nm LED array held 2.5 cm from the treated eye. Each subject served as their own control with only one eye treated. Treatment was applied for a minimum of 2 months with patients given the option to be treated for 9 months. PBM treatment resulted in a significant decrease in NCDME as measured by Spectral-Density OCT (73).

Shen et al. (78) reported on a single arm, non-randomized pilot study involving 21 subjects with CIDME. Subjects were irradiated with 670 nm light at different fluence using custom-designed, slit-lamp microscope mounted Integer laser (Ellex Medical Lasers). Power settings of 25, 100, and 200 mW/cm^2^ were used for 90 seconds, to deliver 2.25, 9, or 18 J/cm^2^ on a 4.5 mm diameter spot, which included a central 1 mm diameter mask to protect the macula through a standard fundus contact lens. Patients received 12 treatments over 5 weeks and follow-up examinations were performed at 2 and 6 months. There was significant reduction of central macular thickness in all groups at both follow-up time points, although there was only a small increase in VA at 2 months, and no change at 6 months, compared to baseline. The authors noted that the macular thickness reduction was more prominent in groups treated with higher doses of 9 and 18 J/cm^2^ (78).

Kim et al. (66) conducted a randomized, multi-center clinical trial enrolling 135 subjects with CIDME and good vision. A total of 69 subjects were treated with 670 nm through an eye patch to deliver 4.5 J/cm^2^ (60 mW/cm^2^, 90 sec) and 66 subjects received low-powered white light as a sham treatment. Patients applied the eye patch 2x daily for 4 months, delivering 1080 J/cm^2^ fluence over the length of the study (~240 treatments). No significant difference between the PBM and sham group in visual VA and central subfield thickness was observed. No serious AEs were reported. While this paradigm of PBM did not lead to beneficial changes in this study, due to earlier positive results in pre-clinical and clinical studies, and considering the limitations of this trial, further studies with different treatment regimens should be considered.

Most recently, Kaymak et al. (47) showed improvements in clinical and anatomical parameters in early-stage DME subjects treated with multiwavelength PBM. Subjects with early-stage DME with good vision (BCVA > 20/25, logMAR > 0.1) were treated with one series of multiwavelength PBM (Valeda: 590 nm, 5 mW/cm^2^; 660 nm, 65 mW/cm^2^; 850 nm; 8 mW/cm^2^) delivered 3x/week over 3-4 weeks for a total of 9 treatment sessions. Following PBM treatment, a reduction in central retinal thickness, resolution of intraretinal fluid, and improvement in diabetic retinopathy severity scale scores were observed. BCVA remained stable following PBM treatment. Approximately 64% of subjects reported subjective improvements in their ocular condition and a decreased negative influence on everyday life activities. No safety issues or concerns related to phototoxicity were observed with follow up conducted out to 16 months.

#### 3.2.3 Additional evidence (exploratory)

Eells et al. (110) conducted a pilot study including 10 subjects with treatment resistant DME. Subjects were randomly placed into the PBM (n=6) or the standard treatment control (n=4) group. Subjects received 670 nm PBM treatment (4.5 J/cm^2^) delivered daily for 3 consecutive days, for 8 weeks. At 24-weeks post-treatment, there was a significant improvement in VA and a reduction of central retinal thickness in the PBM group compared to control.

### 3.3 Amblyopia and myopia

Amblyopia is a developmental visual impairment, most commonly caused by ametropia, a significant refractive difference between the two eyes, or strabismus, a physical misalignment of the eyes. In both cases, the retinal images cannot be fully fused in the visual cortex, resulting in blurred vision. To improve image quality, the brain suppresses one of the retinal images and stunts further visual development in the affected eye. Generally, treatments include the use of glasses, occlusion therapy, and eye drops aimed to weaken the higher performing eye (111). Treatment is only effective if commenced early in childhood. The benefit of PBM to impact retinal health may be of interest in amblyopia and has been studied in one RCT with positive effect (71).

Myopia, commonly referred to as nearsightedness, is a prevalent eye disease with estimated projections to affect nearly 50% of the world’s population by 2050 (112). In the past several years, an abundance of RCTs have been published demonstrating benefits of PBM in children presenting with myopia (64, 65, 67, 113, 114). Overall, studies show exciting and consistent results for positive improvements in myopia outcomes and reduced progression of the condition in children. Following PBM treatment, reductions in spherical equivalent refraction (SER) progression and axial length (AL) are observed. The beneficial mechanisms of PBM in myopia are uncertain but effects at the mitochondrial level, improved choroidal blood perfusion, and dopamine influence are proposed (115). Further details on all RCTs using PBM in myopia have been extensively reviewed elsewhere (113, 115–118).

#### 3.3.1 Clinical evidence

Ivandic and Ivandic (71) conducted a single-blinded, placebo-controlled interventional study with 231 eyes of 178 subjects. Ametropia was observed in 110 eyes, while in 121 eyes, amblyopia was caused by strabismus. Subjects were treated with 780 nm light using a continuous wave semi-conductor laser diode. The macula was treated from 1 cm distance transconjunctivally, in an irradiating spot of 3 mm^2^. Each treatment delivered 0.22 J/cm^2^ and was repeated 3-4 times over a 2-week period with an average overall dose of 0.77 J/cm^2^. The authors found that VA improved in around 90% of subjects, while in the remaining subjects, VA was maintained. Visual improvements were maintained for at least 6 months. The rate of improvement was more pronounced in adolescent subjects, and it also correlated with baseline vision. Of note, amblyopia is largely considered an untreatable condition after the first decade of life. This study enrolled a wide range of subjects with a mean age of 46.8 years (age range of 13-72 years). The authors pose that the underlying effects on cellular function and potentially inter-neuronal communication via promotion of synaptogenesis may be driving beneficial outcomes (71).

Multiple RCTs in children with myopia have been conducted. Xiong et al. (68) conducted a parallel design study with 229 school-aged children subjects divided into 3 study arms. The interventional group used single vision spectacle (SVS) lens and was exposed to 650 nm (2+/-0.5 mW) for 3 minutes, 2x daily, over a 6 month period. Control groups included subjects assigned to use orthokeratology (OK) lens or the SVS lens alone. They examined AL, SER, and sub-foveal choroidal thickness (SFCT) at 1, 3 and 6 months, and found a significant shortening of AL, reduced SER, and increased SFCT in the PBM group compared to control (90). Jiang et al. (67) conducted a multicenter, randomized, parallel-group, single-blind clinical trial that included 264 subjects with 2 study arms. Children were randomized to either wear SVS, or to the intervention group which received PBM using 650 nm (0.29mW through a 4mm pupil aperture for 3 minutes). Treatment was delivered 2x daily for 5 days a week over a 1-year period. In this study the laser diode provided a 0.4 J/cm^2^ single dose, overall delivering 208 J/cm^2^ irradiation fluence over the year. These authors also reported a significant shortening of AL and improved SER progression at 3, 6, and 12 month follow-up times. Neither of these studies recorded any AEs in the structure or function of eye in the PBM groups.

Most recently, Zhou et al. (65) evaluated two consecutive cohorts of 50 subjects following 650 nm PBM (0.35 ± 0.02 mW/cm^2^) delivered 2x per day for 3 minutes with at least a 4-hour interval between sessions, over 12-months. The control group received interventional treatment with SVS only. Stabilization of myopia progression was observed with reductions in SER and AL. A subsequent study by Zhou et al. (64) evaluated 3 differing power levels of 650 nm (0.37 ± 0.02 mW; 0.60 ± 0.2 mW; 1.20 mW) with the same treatment protocol over 6-months. A reduction in SER, AL, and increased SFCT was observed. No effect of power was observed although a trend was in favor of the highest power (1.20 mW) was noted. No serious AEs were noted in either study.

### 3.4 Retinitis pigmentosa

RP is the most prevalent inherited retinal dystrophy, affecting over 1.5 million people globally (119). Due to its genetic background, therapeutic efforts have largely focused on gene therapy (120). As the disease has been linked to a variety of single or combination gene mutations, there is still a paucity of effective treatment options. The common feature of this diverse group of blinding diseases is the loss of rod photoreceptors. In addition, most cases show cone degeneration following the loss of rods (121). Previous studies have shown that the underlying mechanisms leading to the progressive loss of photoreceptors include oxidative damage (122). As PBM has been shown to be effective in reducing free radical production and oxidative damage in the retina, it could offer a possible treatment option.

#### 3.4.1 Preclinical evidence

The therapeutic efficacy and mechanism of action of 670 nm PBM was investigated in a rodent model of RP, the P23H rat (123). In this rodent model of human disease, the transgene is a rhodopsin gene engineered to mimic a mutation that causes an autosomal dominant form of human RP, common in North America. P23H rat pups were treated once per day during the critical period of photoreceptor development with a 670 nm LED array (180 sec treatments at 50 mW/cm^2^; fluence 9 J/cm^2^) (Quantum Devices Inc.). Sham-treated rats were restrained, but not exposed to 670 nm light. Two treatment periods were studied. In the first series, rats were treated from postnatal day (P) 16-20, and the retina was examined at P22 by assessment of mitochondria function, oxidative stress, and cell death. In the second series of studies, rat pups were treated from P10-25. Retinal status was assessed at P30 by measuring photoreceptor function by ERG and retinal morphology by OCT. Treatment with 670 nm light increased retinal mitochondria CcO activity, attenuated photoreceptor cell loss, and improved photoreceptor function. These data suggest that PBM protects photoreceptors in the developing P23H retina, by augmenting mitochondria bioenergetics.

A subsequent study by Gopalakrishnan et al. (124) evaluated 830 nm PBM (180 s; 25 mW/cm^2^; 4.5 J/cm^2^) in the P23H transgenic rat model. P23H rat pups were treated from P10 to P25. Mitochondrial redox state, ERG, OCT, and histomorphometry were assessed at p30 to provide a readout of collective retinal metabolic state, function and morphology. Following PBM treatment, all measures were preserved and PBM protected against disrupted mitochondrial oxidation state observed in the sham-treated animals. Scotopic ERG responses were significantly greater following PBM and imaging and histological assessment showed that the retinal structural integrity was preserved. These findings demonstrated a direct effect of NIR PBM on retinal mitochondrial status in a well-established model of retinal disease. They reveal deleterious effects on retinal bioenergetics resulting in mitochondrial dysfunction, and retinal degeneration following proteotoxic stress. The findings support the potential for targeted therapies that aid in normalizing mitochondrial metabolism for the treatment of retinal degenerative disease.

#### 3.4.2 Clinical evidence (exploratory)

A single case report has been published on the effect of PBM in patients with RP. Ivandic and Ivandic (82) describe a 55-year-old male patient with severe reduction of visual fields to 5°, absent ERG b-wave in both eyes, and a 20/50 baseline VA score (82). His family history was unknown and no information was provided about the genetics of his condition. A continuous wave laser diode emitting 780 nm light was used to deliver 0.4 J/cm^2^ energy fluence (0.333 W/cm^2^ over 40 sec) in 4 treatments over 2 weeks from 1 cm distance on the complete conjunctival surface. The overall energy delivered was 1.6 J/cm^2^. A full recovery of VA to 50/50, and expansion of visual fields to normal perimeter limits bar a mid-peripheral concentric scotoma was observed. The condition was maintained for 5 years. Thereafter, the patient’s retinal function deteriorated to baseline levels. Additional treatments were successful to recover and maintain visual improvements. Further studies with larger patient populations need to be conducted, however, this study demonstrated the potential benefit of PBM in mitigating visual loss in RP.

### 3.5 Retinopathy of prematurity

ROP is one of the leading causes of blindness in infants of the western world. The key risk factors of ROP are prematurity (birth before 31 weeks of gestation) and low birthweight (≤ 1250 g) (125). It is associated with the supplemental oxygen therapy (hyperoxia), used to support premature lungs, at a time when the retinal vasculature is not fully developed. The ensuing retinal hyperoxia suppresses VEGF production, inhibiting retinal vessel growth. Once oxygen therapy is discontinued, the retina becomes hypoxic due to the lack of patent retinal vessels, leading to a rebound increase in production of VEGF and aberrant re-vascularization. The impact of these pathological changes can be devastating to the immature retina. Death of photoreceptor cells and retinal detachment can result in profound visual deficits (126). Current treatments include laser therapy, cryotherapy, and intraocular injection of anti-VEGF agents. Approximately 10% of eyes go blind despite treatment (127). A non-invasive treatment or prevention would revolutionize the management of ROP and therefore it presents a significant clinical opportunity for improving premature healthcare.

#### 3.5.1 Preclinical evidence

As oxidative stress is key in the pathogenesis of ROP, PBM is posed to be a promising treatment option to prevent or mitigate retinal pathologies. Studies using oxygen-induced retinopathy (OIR), the animal model of ROP, have demonstrated that PBM is effective in mitigating retinal pathology in mice and rats. Natoli et al., using 670 nm wavelength at the fluence of 9 J/cm^2^ delivered at a 2.5 cm distance for 3 minutes [WARP 75, Quantum Devices], show significant reductions in retinal vascular pathology (43).

#### 3.5.2 Clinical evidence (exploratory)

Kent et al. (80) conducted a clinical feasibility study to assess the safety of 670 nm light irradiation [Warp 75, Quantum Devices] to treat 28 preterm babies (< 30 weeks’ gestation, and < 1150 g weight) while undergoing supplemental O_2_ treatment in isolettes. This study demonstrated the safety of PBM treatment for preterm babies, with no reported toxicity, skin burns, or other AEs. This was followed by a randomized control trial, with 86 enrolled neonates (69). Babies were treated daily, using the same commercial device, delivering a full body dose of 9 J/cm^2^ at 25 cm distance for 15 minutes, up to 34 weeks corrected age. The study did not show a significant difference in the severity of ROP, or the requirement for laser treatment between the treated and control groups. The authors, however, reported a survival rate of 100% in the PBM-treated group, as opposed to the 89% of controls, although this did not reach statistical significance (69). A prospective observational cohort study performed by Garcia-Serrano et al., evaluated the impact of diode laser treatment in 351 infants with ROP. Following the introduction of laser diode treatment, the 2005-2011 incidence of unfavorable structural outcomes associated with ROP was reduced from 13% to 5.6%. From 2009 to 2012, the incidence of ROP decreased from 55% to 29% (128). Further studies are needed to evaluate PBM effect in ROP.

### 3.6 Central serous chorioretinopathy

Central serous chorioretinopathy (CSCR) is characterized by visual disturbances, detachment of the RPE, and fluid buildup/edema. Visual disturbances include blurred central vision, wavy lines, and color vision distortions. Some patients show irreversible functional and anatomical changes in the retina if spontaneous resolution does not occur. Treatment approaches are commonly laser photocoagulation, photodynamic therapy (PDT) and anti-VEGF ocular injections (1, 2).

#### 3.6.1 Clinical evidence (exploratory)

A report detailing the first case of PBM effect in a patient with CSCR was recently published (52). Following confirmation of CSCR diagnosis, the patient was started on multiwavelength PBM treatment (Valeda: 590 nm, 5 mW/cm^2^; 660 nm, 65 mW/cm^2^; 850 nm; 8 mW/cm^2^) and showed immediate improvements in BCVA and a reduction of fluid build-up in the RPE within 1 week of treatment (3x PBM treatments). Fluid was completely resolved and BCVA score had improved from 55 to 80 ETDRS letters after the full series of PBM treatment. The patient elected to do a repeat PBM treatment series after 6 months and continued to show stable vision with no edema present out to 1-year (52).

### 3.7 Stargardt disease

Stargardt Disease (SD) is a hereditary condition that displays lipofuscin deposits in the paramacular RPE cells. The underlying cause of the disease is a mutation in the ABCA4 gene, resulting in the disruption of the removal of toxic by-products of phototransduction. The degenerative pathological features of SD and loss of central vision may benefit from PBM therapy based on mechanistic insights and relevance.

#### 3.7.1 Clinical evidence (exploratory)

Scalinci et al. (76) conducted a prospective, open-label study with 45 subjects (90 eyes) with stage 1 SD to test the efficacy of 650 nm PBM therapy. Subjects were treated 2x daily (10 minutes) 5 days/week for 12 months. The LED device (Mnemosline glasses, Telea Electronic Engineering) emitted 650 nm light delivered in 10 Hz pulses. They found significant improvements in BVCA, pattern ERG, and microperimetry at 12 months. This study provides foundational evidence and excitement for further studies to confirm PBM benefit in SD.

### 3.8 Leber’s hereditary optic neuropathy

Leber’s hereditary optic neuropathy (LHON) is a mitochondria genetic disorder resulting in acute vision loss in young adults characterized by optic nerve atrophy and the loss of central vision. LHON is caused by primary mutations in mitochondria DNA (mtDNA). The death of retinal ganglion cells in LHON has been attributed to the disruption in mitochondria oxidative phosphorylation combined with increased production of reactive oxygen species. Although treatment options for LHON are limited, clinical trials of gene therapy and mitochondria neuroprotective agents are currently underway (129).

#### 3.8.1 Clinical evidence (exploratory)

A pilot study was conducted in 6 subjects, all affected carriers of the 11778 LHON mutation, exhibiting profound deficits in central vision. The study tested the hypothesis that a brief course of PBM treatment would reduce serum concentrations of the neuronal stress marker, neuron-specific enolase (NSE), and improve visual function. Both eyes were treated with PBM (670 nm; 4 J/cm^2^) once per day for 3 days. Visual function tests showed no detrimental effects of the PBM treatment. Transient improvements in color vision, VA, and visual fields were observed in some, but not all, of the subjects following treatment compared to baseline measures. Importantly, NSE concentrations increased significantly in 2 of the subjects, which potentially is indicative of increased neuronal stress secondary to activation of mitochondria metabolism, therefore the study was discontinued. Based on these findings, the study team concluded that mechanistic investigations of PBM in rodent models of LHON were warranted prior to further clinical trials (130).

### 3.9 Anterior segment of the eye

#### 3.9.1 Corneal trauma/inflammation and orbital implant

Inflammation of the cornea and conjunctiva are common ophthalmic conditions. Corneal foreign body injuries are the second most common ocular injuries (131). Although permanent or severe damage is very rare, the disruption of the corneal epithelium is painful, and deeper damage can result in scar tissue formation.

##### 3.9.1.1 Clinical evidence

A single center, retrospective case series, investigating the effect of PBM on corneal healing following foreign body injury was conducted on 80 eyes of 80 subjects (132). The control group (n=40) received standard care following removal of the foreign body, while the intervention group (n=40) received PBM in addition to standard antibiotics and epithelizing gel treatment. The authors used a custom-built He-Ne laser with changeable spot size to deliver 632 nm light. Patients received trans-corneal treatment of 0.018 J/cm^2^ fluence. Corneal healing time was significantly shortened in the PBM-treated group. Epithelialization was present at 24 hours after the removal, while in the PBM group, healing was complete at 10 hours post-treatment. The authors describe a significant reduction in peri-focal oedema, and conjunctival inflammation in PBM-treated patients.

Another condition, which involves corneal damage is corneal inflammation, keratitis herpetica. The effects of PBM were examined on the rate of corneal healing by the same group, in a study that included 75 subjects (133). Subjects were randomly divided into 3 treatment groups. Two of the groups received standard local antiviral treatment, and the third group was treated by PBM in addition to the local drug administration. Using the laser described above, they delivered a daily dose of 0.054 J/cm^2^ over 3 min. Treatment was used until keratitis fully resolved. Authors found that corneal wound healing was significantly better in the PBM-treated group.

To restore cosmetic appeal of the orbit following enucleation or exenteration, orbital implants are used. The healing of surgical scars is very important in keeping the implant in place. In a study that examined 70 subjects, 35 subjects received PBM in addition to standard postoperative care. The control group only received standard anti-inflammatory drugs (134). Xu et al. applied 632.8 nm light using He-Ne laser postoperatively, delivering 4.5 J/cm^2^ daily for 15 minutes for 10 days. All subjects in the PBM group fully recovered with increased healing time compared to the control group. PBM promoted vascularization of the implant, while attenuated conjunctival redness, and secretion. Normal eyelid activity was also noted.

## 4 Safety considerations

Due to the general benefit of PBM on cellular processes regardless of tissue designation, PBM has been used in a large number of medical indications and thus evaluated for safety across many fields. Preclinical and clinical reports present consistent evidence for no negative effect of PBM in short and long-term evaluations. Furthermore, the wide range of parameters that can be applied (e.g., wavelength, fluence, power, energy, pulse frequency, duration etc.) are not standardized among researchers and clinical devices used, demonstrating the lack of safety concerns voiced among an unusually complex and diverse treatment landscape. Overall, very few side effects have been reported across indications. Most convincing are long-term reports using laser diodes to deliver PBM treatment in cancer applications. PBM is a first-line treatment for oral mucositis due to toxicity induced by chemotherapeutic agents. Extensive follow up has shown no AEs for PBM treatment spanning 15 years or any development of secondary malignancy in treatment protocols for oral mucositis (135). A recent systematic review in oncology reports no significant AEs or tumor safety issues following PBM treatment for the prevention and management of chemotherapy/radiation toxicities (136). Transcranial PBM applications also show no AEs or histological basis for any significant biosafety concerns. Even after longer-term intracranial PBM device implantation, no inflammatory glial response, neuronal degeneration, abnormal upregulation or mitochondrial stress, or impact on surrounding vasculature is observed (137).

No significant AEs have been reported in studies evaluating PBM in ocular indications. These include short-term assessments and studies that span multiple years. PBM use in ophthalmology warrants an increased look at potential hazards to the vulnerable eye tissue. All devices used in the eye should meet the American National Standards Institute (ANSI) and International Electrotechnical Commission (IEC) required safety standard testing for implementing safe laser and LED applications in medical indications. LED and Laser output for all devices used in the eye should be within these acceptable industry standards for eye safety. A recent report on the optical output for two clinical devices confirmed as class 1 laser devices used in ocular indications to deliver PBM showed that 3 minutes of continuous treatment exposure output approached or surpassed thermal and photochemical maximum permissible exposure as dictated by ANSI (138). As utilization of PBM technology continues to grow, efforts are needed to not only further standardize safety and efficacy thresholds for this biotechnology, but to also promote regulation of correctly delivered output across devices. These precautions are paramount to ensure patient safety as well as to reduce contradictory reports of clinical use based on inaccurate output parameters.

As with other light-based treatments, precautions should be exercised in patients who are photosensitive. Laser-based (vs LED) delivery of PBM may warrant additional concerns due to the potential for laser tissue damage/burn. Clinical trial protocols may exclude patients with a presence or history of known light sensitivity to specific wavelengths used for treatment, or if they have a history of light activated central nervous system (CNS) disorders (e.g., epilepsy, migraine). Any use of a photosensitizing agent (e.g., topicals, injectables, oral) prior to treatment without consulting a patient’s physician is not recommended. In addition, caution may be warranted for any open sore(s) that may come into contact with the PBM treatment device, periorbital skin erythema, or patients prone to such conditions with exposure to light.

## 5 Discussion

Further investigation of PBM in a multitude of ophthalmic conditions is warranted, given the underlying biological basis for PBM effect, a favorable safety profile, and the growing number of positive efficacy studies to date. The underlying biological mechanisms of PBM therapy are complex and multidimensional but the growing scientific and clinical evidence demonstrating positive clinical findings in ocular disease has strengthened the use of PBM as a treatment approach for ocular disease and damage. In the field of PBM, we are presented with many variable treatment parameters such as wavelength, treatment duration, and treatment interval, as well as distinct tissue scatter and absorption parameters for each wavelength. In addition, the cellular targets are not equivalent across wavelengths. This provides significant challenges in device design and clinical study protocols, none the less, an increasing number of clinical studies in ocular indications show positive findings following PBM treatment.

Decades of research in the field of PBM has pointed to influence on mitochondrial output as a primary mechanism for the beneficial effects of PBM therapy (1). Experimental studies including *in vivo* and *in vitro* studies and multiple rodent and nonhuman primate models have been instrumental in identifying mechanistic underpinnings related to observed benefits in cellular health and subsequent clinical outcomes after PBM. The accumulating evidence details several positive cellular effects including bioenergetic upregulation, cytoprotection, reductions in inflammatory responses, and general improvement in cellular output and integrity following exposure to PBM (1, 7). From a basic scientific standpoint, the translation of these benefits across multiple indications in the eye is understandable and thus it is not surprising the growing number of studies illustrating positive outcomes in ocular disease and injury following PBM.

Devices designed to deliver PBM are not standardized nor are they typically regulated to ensure consistent and reliable outputs. This is a considerable challenge to both safety and efficacy outcomes, as well as interpretation of study findings. Some devices may deliver a specified irradiance when first illuminated, but power fades markedly by the end of the treatment period. Home devices, which are mostly handheld, LED clusters, or custom-made devices typically have no regulatory approval and may not have undergone extensive beam profiling or output testing as commercially regulated devices. Future guidelines for PBM studies should include standardized testing and reporting among all devices used in clinical evaluations to ensure consistency. The many modifiable factors for PBM treatment and the current lack of regulation on most devices used remains the biggest challenge to the field. This has most likely contributed to inconsistent report findings. The variation in design provides a framework for what works and what doesn’t, which can be used to further optimize treatment approaches moving forward.

The experimental and clinical data to date is most convincing for beneficial effects of PBM in dry AMD, DR/DME, and myopia. PBM in other ophthalmic conditions are also showing early, promising results. Feasibility and larger scale studies are warranted based on exploratory investigations of PBM treatment for RP, ROP, CSCR and SD. Treatment approaches using PBM may be disease specific. Most studies in myopia show benefit using a 2x per day, daily treatment approach spanning 6-12 months. A lack of effect was observed in DME patients treated 2x daily for 4 months (66), however this was in contrast to a pilot study (73) where DME patients were treated 2x daily for 2-9 months and showed beneficial effects. In general, the most robust and consistent findings for PBM in AMD were found in studies that employ an intermittent treatment design (49–51). The treatment protocol may thus differentially impact different disease states and relevant mechanistic actions for the ocular disease. The best treatment strategy for PBM in ophthalmology studies may require further study in specific diseases, but current work provides a starting point for expansion. Research into diagnostic and visual parameters that show predictive value for clinical improvement for patients treated with PBM are also of interest. Of note, ERG may represent a viable tool with predictive capacity for visual outcomes following PBM treatment (76, 99).

While robust clinical findings such as vision gains are desirable and observable benefits to both the patient and physician, the mechanisms of PBM also point to protection and preservation of treated tissue which makes it of importance in degenerative and progressive conditions that will continue to worsen. Treatment of later stage patients such as AMD patients, may require a more preservative mindset in lieu of robust clinical benefit that slows disease progression and maintains the current status of the patient’s eye health without further deterioration. In AMD subjects, a loss of approximately 4 letters per year is observed as the disease progresses (139). When considering treatment for AMD and other ophthalmic degenerative conditions, the overall benefit of PBM should take into account vision losses associated with natural disease progression, as well as to the potential beneficial effects of PBM on both clinical and anatomical outcome measures.

A limitation to studying the effects of PBM in RCTs is the employment of the control arm. Controlled designs with no light therapy delivered would be noticeable to staff and trial participants, thereby removing any potential masking of the study. Studies with a sham treatment group that have reduced the fluence of PBM delivered but not altogether removing it have been trialed. This results in a low dose/high dose design, thereby modeling an active control arm. A lower dose of PBM still may induce benefits, as some PBM-induced activation of photoacceptor molecules is occurring. The LIGHTSITE studies clearly show a small BCVA clinical effect of their sham arm which delivered a 10-100-fold reduction in the fluence of 590 nm and 660 nm and removal of the non-visible NIR 850 nm wavelength. In the two-year LIGHTSITE III trial, sham treatment had a small beneficial effect of a 3-letter BCVA letter gain at 13-months which was reduced to 1-letter gain at 24-months as disease progressed. It was further identified that the sham benefit appeared limited to the patients with best vision and thus suggests that PBM may differentially impact on the stage of disease. Additionally, trials have also used the companion eye in the same patient as a control (i.e., each patient served as their own control with one eye treated and the other eye not treated). This is not recommended as PBM effect may systemically provide benefit to neighboring tissue; therefore, the non-treated eye may also be expected to show some benefit (140).

A recent cross-sectional retrospective study evaluating clinical trial participation found a significant reduction in patients willing to undergo surgical interventions compared to non-invasive approaches involving topical drugs and/or PBM for retinal disease (141). The non-invasive nature of PBM delivery to the eye, coupled with the favorable safety profile noted across studies, provides two significant factors driving the further exploration of this biotechnology. The repeated treatment schedule has been highlighted as possibly cumbersome and a deterrent for patients regardless of the device-dependent in-home or in-office requirement. In general, PBM treatment protocols are < 5 minutes in duration per session. Studies that provide details on compliance show high rates of patient compliance indicating a motivated population willing to pursue repeated PBM treatment approaches for their ocular conditions (50, 51, 66, 79). Additionally, there are no known drug/device treatments, outside of photosensitizing agents, that are projected to interact with PBM which is highly beneficially in aging populations commonly affected by ocular disease.

Overall, dozens of reports now exist detailing the beneficial effects of PBM for treatment in ocular conditions. Interest in the precise methodology and specifications for delivery of the modality will aid in our understanding of the potential clinical impact among different ocular disease states and severity of each respective disease. Additional studies will undoubtedly further define its efficacy and may lead to the development of personalized treatment protocols in managing ophthalmological conditions. While this review points to more research endeavors with PBM in ocular disease and damage, the overwhelming conclusion from many studies indicates that PBM has the potential to improve patient outcomes in a variety of conditions. The non-invasive nature, safety profile and proposed mechanisms of action motivate further research for PBM therapy in the field of ophthalmology.
